# Case report: Acute appendicitis in appendix duplication

**DOI:** 10.1016/j.ijscr.2023.109044

**Published:** 2023-11-14

**Authors:** Jonathon Clymo, Alona Courtney, Emma V. Carrington

**Affiliations:** a Imperial College Healthcare NHS Trust, St Mary's Hospital, Praed Street, London W2 1NY, United Kingdom; b Department of Targeted Intervention, Division of Surgery & Interventional Sciences, University College London, 3rd floor, Charles Bell House, 43-47 Foley Street, London W1W 7TS, United Kingdom; c The Princess Grace Hospital, HCA Healthcare UK, 42-52 Nottingham Place, London W1U 5NY, United Kingdom; d Imperial College London, Division of Surgery, Department of Surgery & Cancer, St Mary's Campus, Praed Street, London W2 1NY, United Kingdom

**Keywords:** Acute appendicitis, Appendix duplication, Emergency surgery, Training, Case report

## Abstract

**Introduction:**

Duplication of the appendix is a very rare presentation. According to the Cave–Wallbridge classification, there are three types of duplicate appendix.

**Presentation of case:**

A 43 year old female presented with classical symptoms of acute appendicitis, with unremarkable inflammatory markers. The diagnosis was confirmed on pre-operative computer tomography (CT). During laparoscopy two tubular structures were identified: one arising from the tenia libera of the caecum adjacent to the terminal ileum and one retrocaecally at the confluence of the teniae. Both structures were excised using a laparoscopic linear stapler. Histopathological analysis demonstrated the accessory structure to be a microscopically unremarkable blind-ended tubular structure. The other specimen demonstrated acute gangrenous inflammation of the appendix. The patient had an uneventful recovery and was discharged home the following day.

**Discussion:**

Appendix duplication is rare; however, failure to recognise it in a patient with acute appendicitis could result in a retained source of sepsis, requiring subsequent re-exploration of the abdomen. The case presented here represents a Type B2 according to the Cave-Wallbridge classification and is the most susceptible to inadvertent error due to having appendixes in both typical and atypical anatomical locations. This case also highlights the probability of this diagnosis being missed on pre-operative CT.

**Conclusion:**

This case report presents a unique opportunity for surgical trainees to review intra-operative laparoscopic images of a duplicate appendix, both to allow them to recognise this pathology if encountered in the future, and to embed the importance of ruling it out with thorough intra-operative examination.

## Highlights

• Appendix duplication is rare; however, it is potentially dangerous in appendicitis.
• Type B2 appendix duplication is the most susceptible to inadvertent error.
• It is crucial to consider rare pathology during routine intraoperative examination.
• Surgical training relies on experientially embedding the possibility of rare cases.

## 1 Introduction

Duplication of the appendix is a very rare presentation with estimated incidence 0.0004 % [1]. According to the Cave–Wallbridge classification, there are 3 types of duplicate appendix: Type A with a single caecum and single appendix with partial duplication, Type B with a single caecum and two appendixes, and Type C with duplication of caecum and appendix [2]. Subtype B1 has symmetrical ‘avian type’ appendixes and subtype B2 has one appendix in the usual place and another further along the taenia coli, making this type of second appendix the most susceptible to being missed [3]. Surgical training from its inception as Halstead's ‘see one, do one, teach one’ to Kolb's more modern learning theory of ‘experience, observation, thinking and action’ requires the trainee to have the richest possible experience of rare pathology in order to consider it intra-operatively [4,5]. We therefore present only the second intra-operative photographs of a duplicate appendix in the literature, and with a substantially different visual appearance to the first [3]. The case has been reported in line with the SCARE criteria 2018 [6].

## 2 Presentation of case

**Fig. 1:**
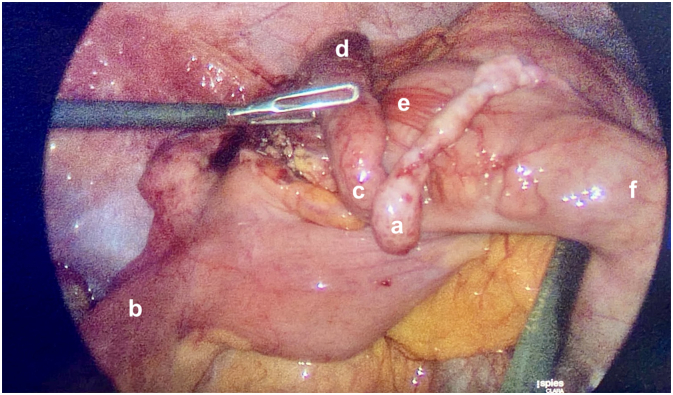
Laparoscopic image showing a non-inflamed duplicate appendix (a), a macroscopically normal tubular structure near the terminal ileum (b) with its base arising from the lateral edge of the tinea coli, as well as a macroscopically inflamed retrocaecal appendix (c - appendix base, d - appendix tip) following mobilisation of the caecum (e) from the lateral abdominal wall. Additional label: f - ascending colon.

**Fig. 2:**
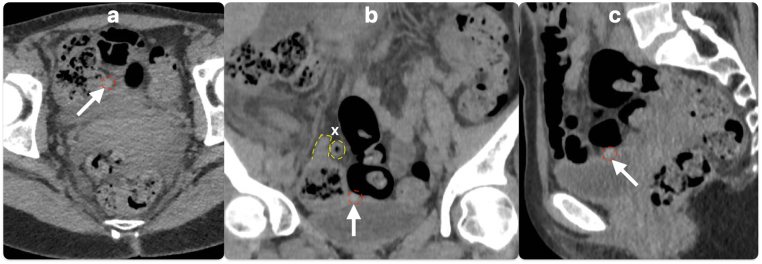
CT images showing axial (a), coronal (b) and sagittal (c) views of the duplicate appendix which was not identified initially. The white arrows indicate the duplicate appendix (outlined in red); the inflamed retrocaecal appendix (x) can also be seen lying superior to the duplicate appendix in panel b (outlined in yellow).

A 43 year old female with no significant past medical history self-presented to the emergency department with a one day history of abdominal pain migrating from the umbilicus to the right iliac fossa, associated with vomiting. Her abdomen was tender in the right iliac fossa with localised guarding. She was afebrile but hypotensive (blood pressure 96/62 mmHg). Her inflammatory markers were within normal limits (white cell count 9.8 × 10^9^/L, neutrophils 7.7 × 10^9/^L, C-reactive protein 10.5 mg/L). Urinalysis revealed isolated microscopic haematuria. A computer tomography (CT) without contrast of the kidney, ureter and bladder was performed to rule out the differential diagnosis of renal colic given the presence of haematuria. This identified an enlarged appendix with surrounding fat stranding consistent with acute appendicitis (Fig. 2). A subsequent laparoscopic appendicectomy demonstrated a tubular structure arising from the tenia libera of the caecum adjacent to the terminal ileum. Although this structure was arising from the caecum, its anatomical location was atypical for an appendix, and it was not inflamed. Mobilisation of the caecum from the abdominal wall revealed an inflamed thickened retrocaecal appendix in a normal anatomical location at the confluence of the teniae (Fig. 1). Both structures were excised using a laparoscopic linear stapler. Histopathological analysis demonstrated the accessory structure to be a microscopically unremarkable blind-ended tubular structure. There was acute ulcerative, suppurative, gangrenous inflammation of the primary appendix. The patient had an uneventful recovery and was discharged home the following day.

## 3 Discussion

Based on the Cave-Wallbridge classification, the case presented here is a Type B2 appendiceal duplication in which one appendix is in the usual anatomical position and the other arises anywhere along the length of tenia coli [2]. This type of duplication is the most commonly reported type in the literature [1]. According to the review by Nageswaran et al [1], 18 % of reported case are Type A [[7], [8], [9]], 6 % are Type B1, 37 % are Type B2 [[10], [11], [12], [13], [14], [15]], 8 % are Type C [16]. Some of the reported complications included appendiceal cancer in the duplex appendix [7], associated small bowel obstruction [13] requiring small bowel resection [17], and most importantly failure to excise one of the appendixes [10] requiring subsequent re-operation [11,18]. To our knowledge, standard practice is to remove a duplicated appendix (if identified at the time of index operation) to avoid potential future diagnostic dilemmas if a patient with a ‘previous appendicectomy’ ever presents with an acute abdomen [15].

Of the duplication types described, Type B2 is the most susceptible to inadvertent error through failure to recognise either that a second atypical appendix exists, or in this case that there remains a native appendix in a classic anatomical location [19]. Type A duplication would be dealt with during the amputation of the single appendix. Type B1, where 2 appendixes arise from either side of the ileocaecal valve, should be identifiable because both are anatomically atypical prompting thorough inspection. Type C, likely to be associated with other congenital abnormalities, would be quickly apparent.

Although appendix duplication is a rare finding in a patient with acute appendicitis, the consequences of failing to recognise it could be grave. In this case of an unperforated retrocaecal appendix with no pus or abscess cavity and unremarkable inflammatory markers, there was a risk of incorrectly identifying the uninflamed duplicate appendix as the source of the patient's symptoms. If the duplicate appendix alone was excised, the source of sepsis would have been left behind. In many cases the diagnosis of appendicitis is made based on history and clinical examination alone, without pre-operative imaging to confirm the diagnosis. Even where pre-operative CT is performed, it is highly possible that the duplication will be missed, as illustrated in this case where it was only identified on a retrospective review of the CT scan (Fig. 2).

## 4 Conclusion

Appendix duplication is a rare anatomical variant; however, it has potentially dangerous consequences in appendicitis. Type B2 with one anatomically typical and one atypical appendix is the most open to intra-operative error. Surgical training relies heavily on experiential learning, yet embedding the possibility of rare pathology upon the surgeon performing routine intra-operative examinations has previously relied on written descriptions. It is therefore essential for trainees to have exposure to this rare case in its richest possible format, with the intra-operative photography and correlated CT scan we present here.

## Ethical approval

This case report is exempt from ethical approval in our institution as it is not a research study.

## Funding

This research did not receive any specific grant from funding agencies in the public, commercial, or not-for-profit sectors.

## Author contribution

Jonathon Clymo: data analysis and interpretation; drafting the article and revising it critically for important intellectual content; final approval of the version to be submitted.

Alona Courtney: conception and design of the study; acquisition of data; revising the article critically for important intellectual content; final approval of the version to be submitted.

Emma V Carrington: conception and design of the study; revising the article critically for important intellectual content; final approval of the version to be submitted.

## Guarantor

Emma V Carrington.

## Research registration number

Not applicable to this case report.

## Informed consent

The patient has given written informed consent for the publication of this article.

## Conflict of interest statement

Nothing to declare
